# Can we achieve justice in health? Lessons from population health, human rights and the FCTC process

**DOI:** 10.18332/tid/138496

**Published:** 2021-07-02

**Authors:** Kelsey Romeo-Stuppy, Dudley Tarlton

**Affiliations:** 1 Action on Smoking and Health, Washington, United States; 2 United Nations Development Programme, Istanbul, Turkey

**Keywords:** tobacco, justice, human rights

*The 18th WCTOH has announced its plan to hold a virtual Leadership Summit on Tobacco Control in 2021. Ahead of the Summit, it is planned to hold a series of open-access webinars to provide on-going access to expert opinion on issues of importance to the Tobacco Control community. The fifth webinar of the series ‘Can we achieve justice in health? Lessons from population health, human rights, and the FCTC process’ was held on 16 March 2021.*

*The webinar presented latest developments in tobacco control with key lessons and recommendations for advancing justice in health. Within the context of treaty frameworks that have been ratified by Parties, the speakers highlighted how to reduce health inequities by identifying opportunities for progressing tobacco control within existing frameworks for population health, sustainable development, human rights, and the WHO FCTC.*

## The intersection between tobacco and social justice

Tobacco and social justice are inextricably intertwined. According to the UN, we uphold the principles of social justice when we promote gender equality, or the rights of indigenous peoples and migrants. We advance social justice when we remove barriers that people face because of gender, age, race, ethnicity, religion, culture, or disability. Tobacco negatively impacts the human rights of many populations, women, children, racial minorities, and others. Tobacco is a barrier to achieving social justice.

## What we can do about it?

The human rights system could and should be utilized as a tool in the fight for social justice and as a tool in ending the tobacco epidemic. Strengthening the ability of health advocates to use an effective human-rights framing of tobacco control efforts is essential. Conversely, it is crucial to embed tobacco control into efforts to strengthen human rights and achieve justice. There are several spheres of justice where tobacco control can and should feature prominently – health justice, of course, but also economic justice, climate justice, and social justice.

For health justice, the COVID-19 pandemic provides urgency to reduce the health vulnerabilities of people who use tobacco. For environmental justice, the toll of tobacco on deforestation, solid waste and water, and soil and air pollution, are well documented and tobacco control offers a cost-effective means of mitigating environmental degradation. In terms of economic justice, joint work by UNDP, the Secretariat of the FCTC and WHO with governments in many countries^1-3^ has documented the disproportionate health and economic benefits that accrue to lower income populations when tobacco control is scaled up, making it a significant accelerator of SDG 10 on reducing inequalities. Gender justice should incorporate tobacco control to address the disproportionate harms to women exposed to tobacco secondhand smoke and the increasing rates of tobacco use among women in some region. Social justice movements should also take into account tobacco control given the long history of the tobacco industry of co-opting movement for social change, such as racial and LGBTI equality, to advance their interests at the expense of the targeted populations.

Tobacco control advocates do not frequently engage in the human rights system, but there are myriad opportunities to effect change at the local, national, regional and international level. Domestically, a tobacco control advocate can employ human rights arguments when encouraging local law makers to create new tobacco control policy, for example, illustrating that smoke-free policies help to ensure the human right to a healthy environment. Internationally, advocates can submit parallel reports to human rights treaty bodies (such as a report on how the children of their country are impacted by tobacco, submitted to the Committee on the Rights of the Child), or even advocate at the Human Rights Council, where tobacco control efforts should be evaluated as part of countries’ Universal Periodic Reviews. There are many opportunities to engage in a human rights-based approach to tobacco control, and advocates should be taking advantage of as many as possible.

## CONCLUSION

A human rights-based approach to tobacco control could be a path towards social justice, and away from the death and disease caused by tobacco.
